# Rational Prescribing Under Pressure: A World Health Organization Indicator and National Medical Commission Compliance Audit With Shift-Based Analysis of Emergency Prescriptions at a Tertiary Care Hospital in North India

**DOI:** 10.7759/cureus.109912

**Published:** 2026-05-30

**Authors:** Baldeep Kaur, Ashish Goel, Bhavneet Bharti, Tushar Vashisht, Saurabh Lanjewar, Bhagwant Singh

**Affiliations:** 1 General Medicine, Dr B.R. Ambedkar State Institute of Medical Sciences, SAS Nagar, IND; 2 Pediatrics, Post Graduate Institute of Medical Sciences and Research, Chandigarh, Chandigarh, IND; 3 Pharmacology, Dr B.R. Ambedkar State Institute of Medical Sciences, SAS Nagar, IND

**Keywords:** emergency department prescriptions, generic drugs, nmc compliance, prescription audit, prescription completeness, rational prescribing, shift-based variation, who core prescribing indicators

## Abstract

Background: Rational prescribing is essential for patient safety and cost-effective healthcare. Good prescription writing is important from a treatment, documentation, and medico-legal point of view. The World Health Organization (WHO) prescribing indicators and the National Medical Commission (NMC) guidelines provide standardized benchmarks to evaluate and monitor prescription quality. However, these are mainly applicable to outpatient department (OPD) setups. The high patient volumes, time constraints, and high-pressure, resource-limited environment might alter doctors' prescribing behavior in emergency settings, particularly in developing countries.

Objectives: This study aims to evaluate prescribing practices in a government emergency department using WHO core prescribing indicators and assess prescription completeness against NMC standards. A secondary objective was to evaluate shift-based variation in prescribing quality.

Methods: This retrospective cross-sectional study analyzed 648 prescriptions collected over one year as part of a quality improvement initiative. WHO prescribing indicators, NMC compliance parameters, and prescription completeness metrics were assessed. Statistical analysis included non-parametric tests, chi-square tests, and multivariate regression models.

Results: A total of 1719 drugs were prescribed, with a mean of 2.65 drugs per prescription. Of these, 1115 (64.9%) were prescribed by generic name, and 1500 (87.6%) adhered to the National List of Essential Medicines (NLEM). Antibiotics were prescribed in 42 (6.5%) prescriptions, whereas injections were used in 585 (90.3%) prescriptions. Documentation gaps were significant, with diagnosis recorded in only 55 (8.5%) and complete prescriber identification in just 5 (0.8%) prescriptions. Only 21 (1.2%) drug entries met conventional completeness criteria; it improved to 960 (55.8%) after adjusting for stat-dose prescriptions. Shift-based analysis revealed a significant decline in diagnostic documentation from morning to night shifts, while vital recording remained relatively stable. After adjusting for age, gender, and total medications, night shift was independently associated with higher odds of injection use and lower odds of antibiotic prescribing as compared to morning. Older age and higher total medications were also independent predictors of injection use.

Conclusion: Prescribing practices in the emergency department showed significant deviations from WHO indicators and NMC standards, particularly in documentation and injection use. While low antibiotic prescribing reflects cautious antimicrobial use, high injection rates and poor documentation highlight areas for improvement. Shift-based variation suggests an “off-hours effect” influencing clinical decision-making. Targeted interventions, including structured prescription formats, audit-based feedback, and shift-specific strategies, are needed to enhance prescribing quality and patient safety.

## Introduction

Rational drug prescribing is a cornerstone of effective healthcare delivery, ensuring patient safety, cost-effectiveness, and minimization of adverse drug events. The World Health Organization (WHO) defines rational drug use as the prescription of medications appropriate to patients' clinical needs, in doses tailored to individual requirements, for an adequate duration, and at the lowest possible cost [1]. Deviations from recommended prescribing practices, including polypharmacy, inappropriate antibiotic use, excessive injection use, and underutilization of generic drugs, contribute to increased healthcare costs, antimicrobial resistance, and adverse drug reactions [2].

To standardize prescribing practices, WHO introduced core prescribing indicators in 1993 [3]. These include the average number of drugs per encounter, percentage of drugs prescribed by generic name, percentage of encounters with antibiotic or injection use, and percentage of drugs prescribed from the Essential Medicines List [4]. Although these indicators are widely applied, they were developed primarily for outpatient and primary care settings, and no emergency department (ED)-specific prescribing benchmarks currently exist. This necessitates cautious interpretation when applying these indicators to emergency care.

In India, the National Medical Commission (NMC), formerly the Medical Council of India (MCI), has established guidelines for prescription writing, including complete prescriber identification, generic prescribing, and use of essential medicines in government healthcare facilities. The MCI (Professional Conduct, Etiquette and Ethics) Regulations, 2002, remain the operative framework [5], while newer NMC regulations introduced in 2023 are currently in abeyance [6]. Despite these guidelines, adherence in routine practice remains inconsistent, particularly in high-pressure environments such as EDs, which represent a distinct clinical setting characterized by high patient volumes, acute presentations, diagnostic uncertainty, and the need for rapid symptom relief, all of which may contribute to deviations from ideal prescribing practices [7]. While several Indian studies have evaluated prescribing patterns in outpatient settings, data from EDs, particularly incorporating both WHO indicators and NMC compliance, remain limited.

Additionally, emerging evidence suggests that clinical decision-making and prescribing quality may vary with time of day, influenced by factors such as fatigue, workload, and staffing patterns, commonly referred to as the “off-hours effect” [8,9]. To our knowledge, such an integrated evaluation of prescribing practices in Indian EDs remains limited.

The present study addresses these gaps through a comprehensive prescription audit in a real-world, high-volume ED. The primary objective was to evaluate prescribing practices using WHO core prescribing indicators and to assess prescription completeness against NMC standards. The secondary objective was to evaluate shift-based variation in prescribing quality across morning, evening, and night shifts. By explicitly contrasting these high-pressure, shift-based emergency workflows with traditional, outpatient-focused benchmarks, this audit uniquely highlights how acute clinical environments may influence prescribing patterns and regulatory compliance. Ultimately, this integrated evaluation offers context-specific insights to inform targeted quality improvement interventions, enhance prescribing practices, and strengthen patient safety and medico-legal accountability.

## Materials and methods

Study design and setting

This retrospective cross-sectional study was conducted in the ED of our government tertiary care hospital. The study involved a retrospective analysis of prescriptions collected as part of an ongoing quality improvement initiative aimed at improving emergency services over a one-year period (February 2025 to February 2026). Prescribing practices were evaluated using WHO core prescribing indicators and NMC prescription standards.

Sample

Approximately 700 prescriptions were collected during the quality improvement initiative, regardless of patient demographics or presenting complaints. Prescriptions were sampled by convenience sampling during this period as part of weekly audit sessions. These sessions were conducted to train medical officers, junior residents, and interns working in emergency, providing them with insights into ideal prescription writing, identification of errors, improving diagnostic reasoning, and emphasizing the need for complete identifier information.

For the present study, a retrospective cross-sectional analysis of these already collected prescriptions was done. Prescriptions in duplicate and those with illegible handwriting were excluded from the analysis. A total of 648 prescriptions were included in the final analysis.

Data collection

Data were extracted from prescription records and entered into a structured data collection sheet. Data extraction was performed using a predefined checklist based on WHO prescribing indicators and NMC prescription-compliance parameters. No patient identifiers or personally identifiable information (PII) were entered in the structured data collection sheet. Variables recorded included patient demographics (age, gender), visit timings (morning, evening, night), clinical documentation (chief complaints, history, examination findings, diagnosis, follow-up instructions), prescriber identification (doctor name, qualification, registration number, stamp, signature), and medication details (drug name, dosage, frequency, duration, route of administration). Each drug was further classified as generic or brand name, antibiotic or non-antibiotic, included in the National List of Essential Medicines (NLEM) or not, and by the route of administration (oral, injectable, or topical). Fixed-dose combinations (FDCs) were also identified.

Statistical analysis

Prescriptions were evaluated against WHO core prescribing indicators and NMC compliance parameters. Data were analyzed using Python (Python Software Foundation, Wilmington, Delaware, USA). Continuous variables were expressed as mean ± standard deviation and median (interquartile range), while categorical variables were presented as frequencies and percentages. The Kruskal-Wallis test for comparisons across multiple groups (e.g., age groups and shifts). The chi-square test was used for categorical variables.

Multivariate logistic regression was performed for binary outcomes (injection use and antibiotic use), including shift with morning taken as the reference, age, and total number of medications as predictors, with results reported as odds ratios (ORs) and 95% confidence intervals. Multivariate linear regression (ordinary least squares) was performed for continuous outcomes (percentage of generic prescribing and number of drugs per prescription), with results reported as beta coefficients and 95% confidence intervals. A p-value < 0.05 was considered statistically significant.

## Results

Patient demographics

A total of 648 prescriptions were analyzed. The mean patient age was 38.4 ± 18.8 years. The number of males was 339 (52.3%). The patient distribution across shifts was comparable. Detailed patient demographics and visit distribution are presented in Table 1.

**Table 1:** Patient Demographics and Visit Characteristics (N = 648)

Variable	Category	Value
Age (years)	Mean ± SD	38.4 ± 18.8
	Median (IQR)	35 (25–50)
	Range	0–101
Age group	0–18 years	68 (10.5%)
	19–40 years	328 (50.6%)
	41–60 years	153 (23.6%)
	>60 years	99 (15.3%)
Gender	Male	339 (52.3%)
	Female	309 (47.7%)
Visit shift	Morning	197 (30.4%)
	Evening	257 (39.7%)
	Night	194 (29.9%)

WHO core prescribing indicators

The mean number of drugs per prescription was 2.65 ± 1.59. There were a total of 1719 drug entries, and the drugs prescribed with a generic name were 1115 (62.9%). Antibiotics were prescribed in 42 (6.5%) prescriptions, while injections were used in 585 (90.3%) prescriptions. NLEM compliance was 1500 (87.6%) out of the total drug entries. A comparison of observed prescribing practices with WHO reference indicators is summarized in Table 2.

**Table 2:** Comparison of Observed Prescribing Practices With WHO Core Prescribing Indicators Reference ranges mentioned have been derived from WHO methodology and pooled OPD studies; they must be interpreted with caution in emergency settings. NLEM: National List of Essential Medicines; WHO: World Health Organization; OPD: outpatient department

Indicator	Observed	Ideal	Deviation	Interpretation
Drugs per prescription	2.65 ± 1.59	1.6–1.8	High	Polypharmacy
Generic prescribing (%)	1115 (64.9%)	100%	Low	Suboptimal
Antibiotic encounters (%)	42 (6.5%)	20.0%–26.8%	Low	Conservative Use
Injection encounters (%)	585 (90.3%)	13.4%–24.1%	High	Excessive
NLEM drugs (%)	1500 (87.3%)	100%	Low	Suboptimal

Prescription completeness audit

**Figure 1:**
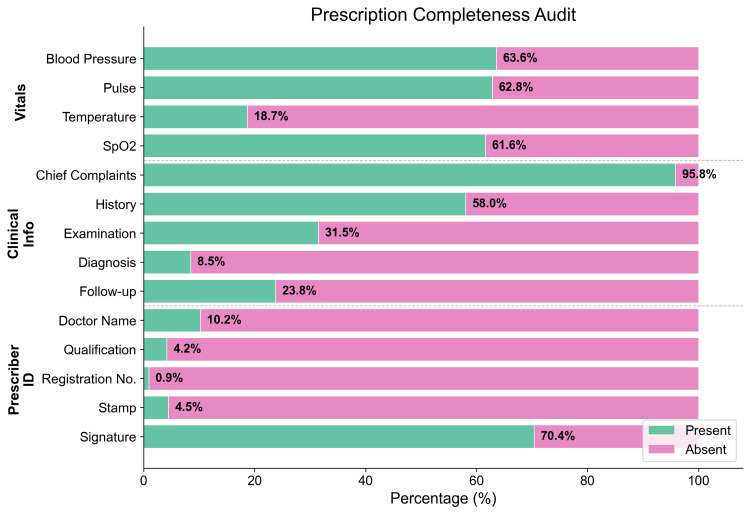
Prescription Completeness Audit: Proportions of Prescriptions With Documented Vitals, Clinical Information, and Prescriber Identification Parameters

Documentation of chief complaints was high (621 (95.8%)); however, key clinical documentation elements were infrequently recorded, with diagnosis documented in only 55 (8.5%) prescriptions and examination findings in 204 (31.5%) prescriptions. Prescriber identification was critically deficient, with only 5 (0.8%) prescriptions being fully compliant as per NMC mandates. Details of clinical documentation and prescriber identification are presented in Table 3 and Figure 1.

**Table 3:** Clinical Documentation and Prescriber Identification Completeness According to NMC Guidelines (N = 648) NMC: National Medical Commission

Domain	Parameter	Result
Vitals	Vitals recorded (any)	484 (74.7%)
	Blood pressure	412 (63.6%)
	Pulse	407 (62.8%)
	Temperature	121 (18.7%)
	SpO₂	399 (61.6%)
Clinical documentation	Chief complaints	621 (95.8%)
	History	376 (58.0%)
	Examination findings	204 (31.5%)
	Diagnosis	55 (8.5%)
	Follow-up instructions	154 (23.8%)
Prescriber identification	Doctor name	66 (10.2%)
	Qualification	27 (4.2%)
	Registration number	6 (0.9%)
	Stamp visible	29 (4.5%)
	Signature present	456 (70.4%)
	Fully identified	5 (0.8%)

Regarding medication details, overall prescription completeness was low: only 21 (1.2%) drugs had documentation of all four essential parameters (dosage, frequency, duration, and route). However, given that a large proportion of drugs were prescribed as single (“stat”) doses, an adjusted analysis revealed that the completeness improved to 960 (55.8%) drug entries. Drug specification and stat-adjusted completeness are summarized separately in Table 4.

**Table 4:** Drug Prescription Completeness and Stat-Dose-Adjusted Analysis (Total Drugs = 1719)

Domain	Parameter	Result
Drug specification	Dosage specified	1344 (78.2%)
	Frequency specified	1486 (86.4%)
	Duration specified	165 (9.6%)
	Route specified	1686 (98.1%)
	% drugs fully specified (all 4)	21 (1.2%)
Stat-dose analysis	Total stat doses, n (%) of all drug entries	1086/1719 drugs (63.2%)
	Stat doses with dosage + route, n (%) of all stat doses	879/1086 (80.9%)
	Non-stat drugs with dosage + route + frequency, n (%) of all non-stat drug entries	81/633 (12.8%)
	Stat-adjusted completeness, n (%) of all drug entries	960/1719 drugs (55.8%)

Drug prescribing details

A total of 1719 drugs were prescribed across 648 prescriptions. Generic drug count was 1115 (64.9%). Proton pump inhibitors (PPIs), analgesics, and antiemetics were the most commonly prescribed therapeutic classes, reflecting predominantly symptomatic management in the ED. FDCs accounted for 114 (6.6%) drug entries. Detailed drug utilization patterns, including the distribution of therapeutic categories (proportion of prescriptions, n=648) and the most commonly prescribed drugs (proportion of total drug entries, n=1719), are presented in Tables 5, 6, respectively.

**Table 5:** Distribution of Drug Categories Across 648 Prescriptions PPI: proton pump inhibitor; NSAID: non-steroidal anti-inflammatory drug; IV: intravenous

Drug Class	Frequency (n)	Prescriptions (%)
PPI	326	50.3%
Analgesic	219	33.8%
Antiemetic	188	29.0%
Antispasmodic	144	22.2%
NSAID	123	19.0%
IV Fluid	70	10.8%
Electrolyte	64	9.9%
Analgesic/Antipyretic	63	9.7%
Antibiotic	42	6.5%
Diuretic	41	6.3%
Vaccine	40	6.2%
Corticosteroid	38	5.9%
Others (≥1 of 75 rarer classes)	244	37.7%

**Table 6:** Top 10 Most Commonly Prescribed Drugs (Out of Total Drug Entries; N=1719) ORS: oral rehydration solution

Drug (Generic Name)	Frequency (n)	Percentage (%)
Pantoprazole	351	20.4%
Ondansetron	174	10.1%
Diclofenac	169	9.8%
Paracetamol	138	8.0%
Hyoscine butylbromide	80	4.7%
ORS	67	3.9%
Normal saline	63	3.7%
Drotaverine	59	3.4%
Tetanus toxoid	46	2.7%
Furosemide	40	2.3%

Analysis of brand-name prescribing revealed that 601 (35.0%) drug entries were by the brand name. The most commonly prescribed brands were those for ondansetron (Emeset/Emset/Ondem; n = 92 (5.4%), hyoscine butylbromide (Buscopan; n = 80 (4.7%), and furosemide (Lasix; n = 40 (2.3%). The details of other commonly used brands are shown in Table 7.

**Table 7:** Brand-Generic Mapping of Commonly Prescribed Drugs

Brand Name	Generic Name	Drug Class	Frequency (n)
Emeset/Emset/Ondem	Ondansetron	Antiemetic	92
Buscopan	Hyoscine butylbromide	Antispasmodic	80
Lasix	Furosemide	Diuretic	40
Drotin	Drotaverine	Antispasmodic	38
Budecort	Budesonide	Corticosteroid	32
Rantac	Ranitidine	H2 Blocker	20
Duolin	Levosalbutamol + Ipratropium	Bronchodilator	16
Tranexa	Tranexamic acid	Antifibrinolytic	13
Telma	Telmisartan	Antihypertensive	11
Dolo	Paracetamol	Analgesic/Antipyretic	3

Shift-based variation in prescribing quality

Prescriptions were distributed across three shifts: morning (197 (30.4%)), evening (257 (39.7%)), and night (194 (29.9%)). Gender distribution did not differ significantly across shifts (chi-square = 0.23, p = 0.891), but age distribution did differ significantly (Kruskal-Wallis H = 15.58, p = 0.0004; age-group chi-square = 22.30, p = 0.0011). Elderly patients (>60 years) presented disproportionately higher during morning shifts, while pediatric and young-adult patients were more evenly distributed.

Most WHO indicators did not differ significantly across shifts, except for injection use. Both the proportion of encounters involving any injection (chi-square = 6.68, p = 0.035) and the percent injectable drugs per prescription (Kruskal-Wallis H = 6.02, p = 0.049) differed significantly.

NMC compliance parameters showed significant variation across shifts, as depicted in Table 8.

**Table 8:** Shift-Based Variation in NMC Compliance Parameters NMC: National Medical Commission

NMC Parameter	Morning	Evening	Night	Chi-square	P-value	Cramer's V	Significant (p<0.05)
Diagnosis documented	25 (12.7%)	20 (7.8%)	10 (5.2%)	7.42	0.0245	0.1070	Yes
Vitals recorded	154 (78.2%)	197 (76.7%)	133 (68.6%)	5.65	0.0593	0.0934	No
Chief complaints documented	186 (94.4%)	248 (96.5%)	187 (96.4%)	1.43	0.4900	0.0469	No
Follow-up instructions	39 (19.8%)	73 (28.4%)	42 (21.6%)	5.24	0.0726	0.0900	No
Prescriber fully identified	4 (2.0%)	1 (0.4%)	0 (0.0%)	6.08	0.0479	0.0968	Yes
% drugs fully specified	0 (0%)	0 (0%)	0 (0%)	H=7.04	0.0297	eps-sq=0.0078	Yes

Diagnosis documentation varied significantly by shift (chi-square = 7.42, p = 0.024, Cramer's V = 0.107), with morning shifts having the highest rate (25 (12.7%)) and night shifts the lowest (10 (5.2%)). Prescriber identification also varied significantly across shifts (chi-square = 6.08, p = 0.048, Cramer's V = 0.097), with complete prescriber identification highest in the morning shifts (4 (2.0%)) and absent during night shifts (0 (0.0%)).

**Figure 2:**
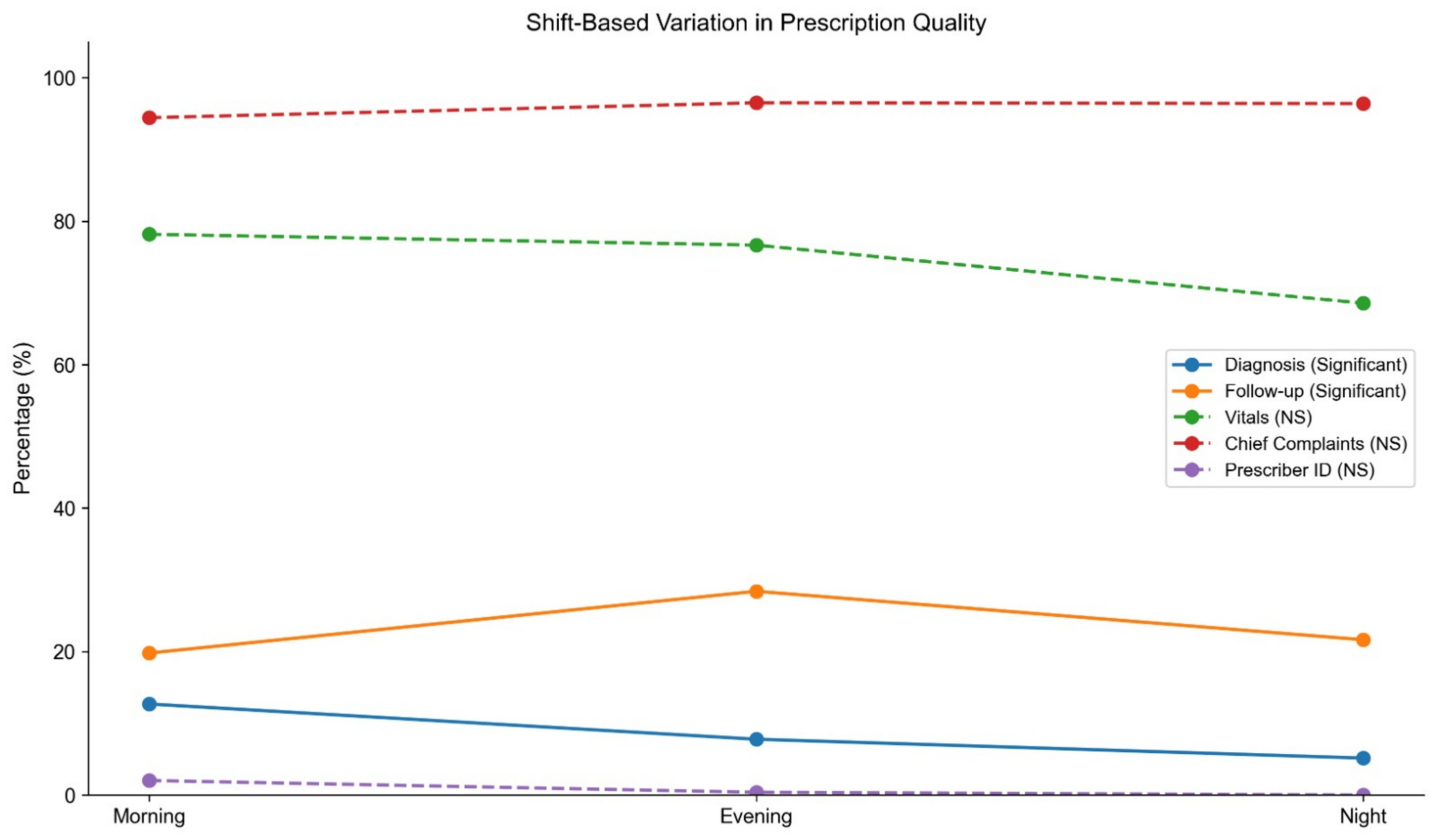
Shift-Based Variation in Prescription Quality Shift-based variation in prescription quality showing differences in diagnosis documentation, follow-up instructions, and key documentation parameters across morning, evening, and night shifts. Parameters with statistically significant differences are highlighted in continuous lines, while those not statistically significant are shown in dotted lines.

The proportion of drugs fully specified differed significantly across shifts (Kruskal-Wallis H = 7.04, p = 0.030, epsilon-squared = 0.0078). In contrast, vitals recording (p = 0.059), chief complaints documentation (p = 0.490), and follow-up instructions (p = 0.073) did not vary significantly across shifts. Complete prescriber identification remained critically low across all shifts (0.0%-2.0%). Shift-based variation in prescription quality is illustrated in Figure 2.

Subgroup analysis

No statistically significant differences were observed between genders for any WHO prescribing indicator.

Age-based analysis revealed significant variation in prescribing patterns. Generic prescribing differed significantly across age groups (Kruskal-Wallis test, p = 0.004), and so did the injection use (p < 0.001). Pediatric patients (0-18 years) had lower rates of injection use and higher rates of generic prescribing compared to adult age groups.

Multivariate regression analysis

Multivariate logistic regression analysis demonstrated that, after adjusting for age, gender, and total medications, the night shift was independently associated with higher odds of injection use compared with the morning (OR 2.78 (1.26-6.14), p = 0.011). Older age was also independently associated with increased odds of injection use (OR 1.03 (1.01-1.04), p = 0.002), as was a higher number of medications (OR 1.27 (1.04-1.55), p = 0.018). For antibiotic use, the total number of medications prescribed was the strongest predictor (OR 2.66 (2.09-3.39), p < 0.001), whereas night shift was associated with lower odds of antibiotic prescribing (OR 0.25 (0.08-0.78), p = 0.017).

Multivariate linear regression analysis showed that increasing age was associated with lower generic prescribing (β = -0.25 (-0.41--0.10), p = 0.001), whereas a higher number of medications was associated with increased generic prescribing (β = 1.81 (0.04-3.58), p = 0.045). Further, the shift was not a significant predictor of generic prescribing. Full regression results are presented in Tables 9, 10.

**Table 9:** Multivariate Regression Analysis of Factors Associated With Prescribing Outcomes - Logistic Regression (Binary Outcomes)

Variable	Injection Use OR (95% CI)	P-value	Antibiotic Use OR (95% CI)	P-value
Age	1.03 (1.01–1.04)	0.002	1.00 (0.98–1.02)	0.862
Total medications	1.27 (1.04–1.55)	0.018	2.66 (2.09–3.39)	<0.001
Evening shift	1.01 (0.56–1.84)	0.966	0.49 (0.21–1.13)	0.094
Night shift	2.78 (1.26–6.14)	0.011	0.25 (0.08–0.78)	0.017
Gender (male)	0.72 (0.42–1.24)	0.242	1.82 (0.85–3.92)	0.124

**Table 10:** Multivariate Regression Analysis of Factors Associated With Prescribing Outcomes - Linear Regression (Continuous Outcomes) Multivariate regression analysis showing predictors of injection use, antibiotic use, generic prescribing, and number of drugs per prescription. Odds ratios (ORs) are reported for logistic regression and beta coefficients (β) for linear regression. Age and total number of medications were significant predictors, while shift was not significantly associated with outcomes.

Variable	Generic Prescribing β (95% CI)	P-value	Drugs per Prescription β (95% CI)	P-value
Age	-0.25 (-0.41–0.10)	0.001	-0.01 (-0.01–0.00)	0.081
Total medications	1.81 (0.04–3.58)	0.045	-	-
Evening shift	4.60 (-2.19–11.40)	0.184	0.11 (-0.19–0.41)	0.460
Night shift	-3.20 (-10.52–4.11)	0.390	0.00 (-0.32–0.33)	0.978
Gender (male)	-1.94 (-7.55–3.67)	0.497	-0.03 (-0.28–0.21)	0.794

## Discussion

This study reveals significant deviations from both WHO prescribing indicators and NMC compliance standards in an ED setting, highlighting critical gaps in prescribing practices and documentation quality and thereby defining the areas requiring targeted intervention.

The average number of drugs per prescription (2.65) exceeded the WHO reference range, indicating polypharmacy. While this may reflect the need for rapid symptomatic relief in acute care, it raises concerns regarding potential drug interactions, adverse effects, and medication errors. Similar trends have been reported in other Indian and other South Asian studies, suggesting that polypharmacy remains a widespread issue across healthcare settings [10,11].

Drugs with generic prescriptions (64.9%) fell below the WHO ideal and the NMC mandate of 100% in government healthcare settings. This is in line with reports from other Indian studies, where generic prescribing rates typically range between 30% and 70% [12]. Low generic prescribing elevates healthcare costs for patients and the healthcare system, despite the proven bioequivalence and cost-effectiveness of generic medications. However, the relatively higher NLEM compliance (87.6%) and minimal variability in brand names suggest that this pattern is more likely driven by prescriber habit and familiarity rather than drug availability constraints.

Older patient age was associated with lower generic prescribing, which might be clinically plausible, as elderly patients may require specific branded formulations or have prior prescriptions that influence continuation of brand-name drugs. The finding that the higher total medications were associated with higher generic prescribing might be due to the large number of generic drugs being available as single-component drugs rather than FDCs, thereby augmenting the total drug count in the prescription.

The most striking finding was the very high rate of injection use (90.3% of the total prescriptions), which showed the highest deviation from the reference range of 13.4-24.1% reported in pooled outpatient studies from developing countries [13]. It is important to note that this reference range is not a WHO-defined ideal but is derived from aggregated outpatient data, and no ED-specific benchmarks currently exist. In the ED setting, increased use of parenteral therapy may be clinically justified in conditions such as trauma, severe pain, asthma exacerbations, or altered consciousness, where rapid onset of action and inability to tolerate oral medications necessitate injectable treatment. However, the magnitude observed suggests that factors beyond clinical necessity may contribute, including physician preference and patient expectations favoring injections as more effective. Excessive injection use is concerning due to the increased risk of complications such as infections and anaphylaxis, as well as higher healthcare costs.

The higher injection rates observed during night shifts may further reflect contextual factors such as greater severity of presentations, reduced staffing and supervision, and limited access to diagnostic support, prompting reliance on parenteral therapy for immediate symptom control. Additionally, physician fatigue during prolonged night duty hours may lower the threshold for choosing parenteral over oral alternatives, as injectable treatments provide more rapid and visible effects [14].

In contrast, the antibiotic prescribing rate was well below the reference range of 20-26.8% reported in outpatient settings. This likely reflects the symptomatic nature of most ED presentations, where initial management often prioritizes stabilization and symptomatic relief rather than immediate initiation of definitive antimicrobial therapy. Emergency physicians frequently serve as the first point of contact, and the decision to start antibiotics is deferred until further clinical evaluation, laboratory investigations, or consultation with senior physicians is obtained. The high patient turnover and diagnostic uncertainty in the early stages of assessment further support a watchful approach before initiating antibiotics. This pattern aligns with antimicrobial stewardship principles and may be considered a positive finding. In multivariate analysis, night-shift presentations showed significantly lower odds of antibiotic prescribing than morning (OR 0.25 (0.08-0.78), p = 0.017), consistent with deferred definitive antibiotic decisions under time pressure and diagnostic uncertainty at night.

A key finding of this study is the substantial deficiency in prescription completeness. While basic documentation, such as chief complaints (621 (95.8%)) and vital parameters (484 (74.7%)), was frequently recorded, critical elements, including diagnosis (55 (8.5%)) and examination findings (204 (31.5%)), were markedly under-documented. This pattern suggests a shift toward symptom-based rather than diagnosis-driven management, likely influenced by time constraints, workload, and diagnostic uncertainty in emergency settings. As seen in other studies, while this approach may be appropriate in certain acute scenarios, it raises concerns regarding clinical reasoning, continuity of care, documentation standards, and appropriateness of treatment decisions [15].

With respect to drug-related documentation, only 21 (1.2%) drug entries met the conventional criteria for completeness, indicating a major gap in prescription quality. However, this estimate requires interpretation in the context of emergency settings where a significant proportion of medications were prescribed as single (“stat”) doses. When a stat-adjusted approach was applied, completeness improved substantially to 960 (55.8%). This highlights an important methodological consideration that traditional completeness metrics may underestimate actual prescribing quality in emergency settings and should be adapted accordingly.

An important concern identified in this study was the low rate of complete prescriber identification, with only five (0.8%) prescriptions containing all the details as mandated by NMC guidelines. This has significant implications from both accountability and medico-legal perspectives. Incomplete identification limits traceability of clinical decisions, weakens the integrity of medical records, and reduces the effectiveness of audit and feedback processes - an issue also noted during our quality improvement sessions. Proper prescriber identification is essential for attributing responsibility, reviewing decisions, and ensuring appropriate follow-up in case of errors or adverse outcomes. From a legal perspective, incomplete prescriptions may compromise their validity as documentation of care, potentially affecting both patient rights and clinician protection [16].

This deficiency likely reflects a combination of high workload, time constraints, and inadequate emphasis on documentation practices during training. However, given its implications, improving prescriber identification should be considered a priority area for intervention. Practical measures such as standardized prescription formats, mandatory documentation fields, use of stamps or electronic prescribing systems, and regular audits with feedback may help address this gap effectively [17].

Shift-based variation in prescription quality, as depicted earlier in Figure 2, demonstrated a decline in diagnostic documentation from morning to night shifts, supporting the concept of the “off-hours effect” [18]. The lowest rates observed during night shifts may reflect increased workload, reduced staffing, and physician fatigue. In contrast, follow-up instructions and vital sign documentation remained relatively stable across shifts, suggesting that routine protocols are less affected by temporal factors compared to higher-order clinical decision-making processes. These findings underscore the need for shift-specific quality improvement strategies, including structured documentation templates, mandatory fields for key parameters, targeted night-shift audits, and measures to address physician fatigue.

Comparison with other Indian studies shows both similarities and key differences across settings. A study from Pandit Bhagwat Dayal Sharma Post Graduate Institute of Medical Sciences (PGIMS) Rohtak (2024) reported higher antibiotic use (20.59%) and much lower injection use (1.15%) compared to our findings (6.5% and 90.3%, respectively) [17]. Studies from tertiary centers in Puducherry and Coimbatore report similar polypharmacy and suboptimal generic prescribing but lower injection use and higher antibiotic prescribing [19,20].

Notably, similar patterns are reported internationally. A study by Desalegn AA demonstrated persistent deviations from WHO prescribing standards, including polypharmacy and irrational drug use [21]. Importantly, evidence from comparable studies highlights significant gaps in prescription documentation, particularly inadequate recording of clinical indications and incomplete treatment details, which contribute to inappropriate prescribing and medication errors. These findings underscore that deficiencies in prescription completeness are not confined to the Indian context but are characteristic of many low- and middle-income health systems [22]. Furthermore, such irrational and incomplete prescribing practices appear to be exacerbated in high-pressure clinical environments [23].

Taken together, these findings suggest that while polypharmacy and incomplete generic prescribing are common across healthcare settings, the combination of very high injection use and low antibiotic prescribing appears to be characteristic of emergency care. These differences are likely driven by variation in case mix, with EDs managing more acute, non-infectious presentations such as trauma and severe pain. At the same time, the persistence of polypharmacy and suboptimal generic prescribing across all levels of care underscores the need for broader, system-level interventions to promote rational drug use.

Overall, these findings suggest that while certain fundamental aspects of prescription writing are routinely followed, there are significant gaps in higher-order documentation and regulatory compliance. Targeted interventions, including structured prescription formats, mandatory documentation fields, and audit-based feedback mechanisms, are essential to address these deficiencies and improve both clinical and medico-legal quality of care.

Regulatory and policy implications

The findings of this study are particularly relevant in the context of evolving regulatory expectations in India. Increasing judicial and institutional emphasis on generic prescribing, accountability, and proper documentation reflects a shift toward stricter adherence to prescribing standards [6]. Although enforcement remains variable, these expectations underscore the importance of compliance. In this setting, the deficiencies identified - especially in prescriber identification and documentation - are concerning and warrant focused attention.

An important methodological consideration is the applicability of WHO prescribing indicators to emergency care. These indicators were originally developed for outpatient settings, and their direct use in EDs should be interpreted with caution. Given the higher acuity of illness and frequent reliance on parenteral therapy, some deviations - particularly in injection use - may represent appropriate clinical adaptation rather than irrational prescribing. This highlights the need for developing context-specific benchmarks for EDs.

Strengthening adherence to regulatory standards through structured audits, targeted training, and improved documentation systems is essential to enhance patient safety and reduce medico-legal risk.

Strengths and limitations

This study has several strengths. It includes a relatively large sample size and a comprehensive evaluation of both WHO prescribing indicators and NMC compliance parameters using standardized assessment criteria. Its integration within an ongoing quality improvement initiative adds practical value, as regular audit sessions also served as structured teaching opportunities for medical officers, junior residents, and interns.

The inclusion of shift-based analysis adds a novel dimension by exploring temporal variation in prescribing practices, an area that has been relatively underexplored in Indian emergency settings. The use of stat-dose-adjusted completeness further provides a more context-appropriate assessment of prescription quality in emergency care settings.

However, certain limitations must be acknowledged. First, the retrospective design relied on information recorded in prescriptions, which may not fully reflect the underlying clinical decision-making process. Second, the study focused on prescription completeness and documentation/compliance parameters and did not assess clinical appropriateness, indication-wise justification of prescribed drugs, treatment effectiveness, adverse drug events, or patient outcomes. Third, being a single-center study, the findings may not be generalizable to other settings. Fourth, because this was a retrospective analysis of prescriptions collected during a quality improvement initiative rather than a real-time observational audit, contextual factors such as overcrowding, staffing, and workload could not be directly assessed. Finally, the WHO prescribing indicators used for comparison were originally developed for outpatient settings and may not fully capture ED prescribing dynamics, necessitating cautious interpretation, particularly for injection use and prescription completeness.

## Conclusions

This prescription audit demonstrated significant deviations from WHO prescribing indicators and poor compliance with NMC prescription standards in a government ED. Key concerns included polypharmacy, suboptimal generic prescribing despite regulatory mandates, incomplete documentation of diagnosis and drug details, high injection use, and near absence of prescriber identification. These findings highlight the need for targeted system-level interventions, including standardized prescription formats, regular clinician training, periodic prescription audits with feedback, ED-specific prescribing guidelines, and stronger administrative oversight. Shift-focused measures, particularly during night shifts, may further help reduce documentation gaps and fatigue-related prescribing errors. Overall, improving prescription completeness and rational drug use in high-volume emergency settings is essential to enhance patient safety, accountability, and quality of care.
